# Supplementary figures and images for: Genome wide CRISPR screen for Pasteurella multocida toxin (PMT) binding proteins reveals LDL Receptor Related Protein 1 (LRP1) as crucial cellular receptor
Source: PLoS Pathog. 2022 Dec 14;18(12):e1010781. doi: 10.1371/journal.ppat.1010781 (PMC9797058; doi:10.1371/journal.ppat.1010781)

**A****PMT<sub>C1165S</sub>**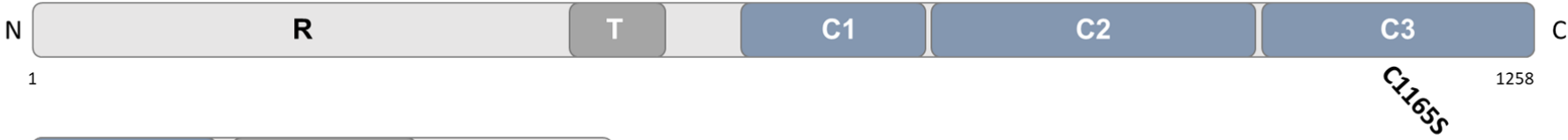**B****DT**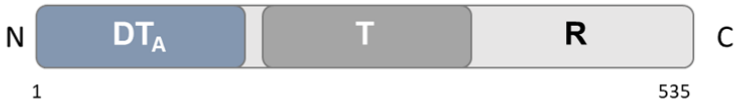**C****PMT<sub>C1165S</sub>-DT<sub>A</sub>**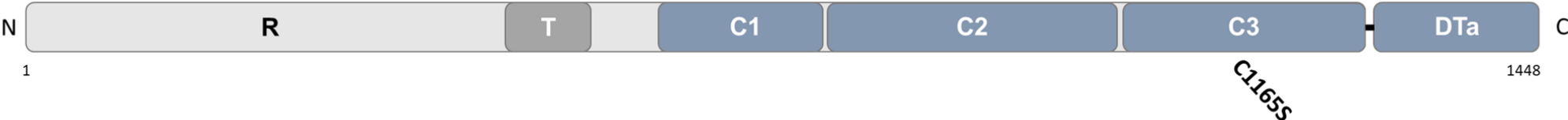

Supplement: S1 Fig — A: The Pasteurella multocida toxin (PMT) is composed of 5 domains: R: receptor binding domain, T: translocation domain containing two hydrophobic helices which are necessary for insertion into the endosomal membrane, C1-3: catalytic domain. C3 encodes for the deamidase domain. Mutation of C1165 to serine leads to a catalytically inactive toxin. B: Diphtheria toxin (DT) is composed of three domains: DTa is the catalytic domain, T: translocation domain, R: receptor binding domain. C: The fusion protein PMT(C1165S)DTa is composed of the catalytic inactive mutant of PMT and a c-terminally added catalytic domain of diphtheria toxin (DTa). (PDF) [file ppat.1010781.s001.pdf]

A

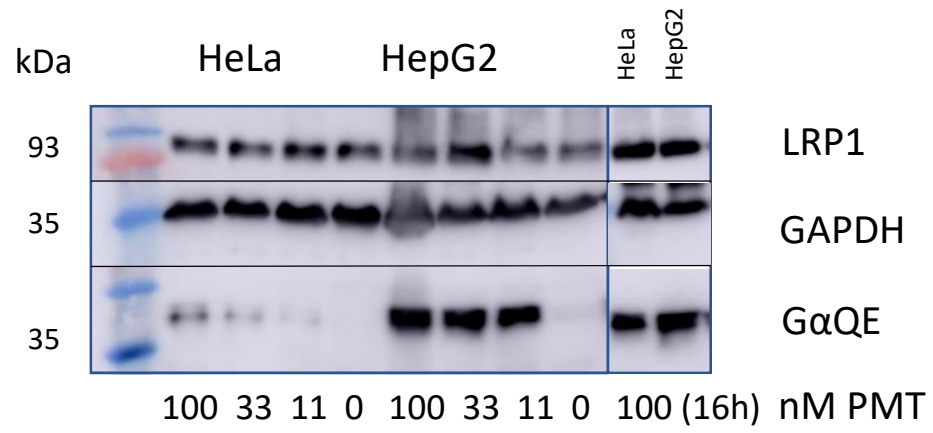

B

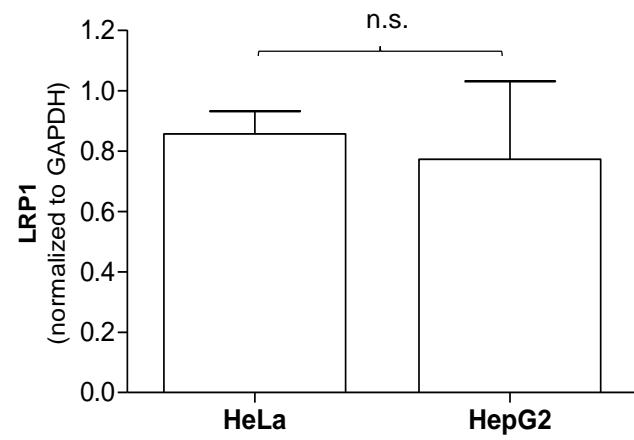

C

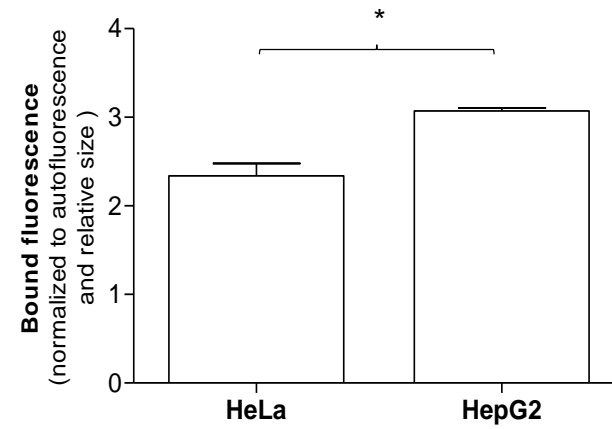

Supplement: S3 Fig — HeLa and HepG2 cells were treated with the indicated concentrations of PMT for 2 h, or with 100 nM PMT for 16h as indicated, washed and lysed. A: Lysates were analyzed for toxin-induced modification of GαQ, LRP1 and GAPDH by Western-blotting. Shown is an example of 4 independent experiments. B: Quantification of the amount of LRP1 normalized to GAPDH in HeLa and HepG1 cells. C: HeLa and HepG2 cells were incubated with 1 μM Alexa 488 labeled PMT and washed. Cell bound fluorescence was analyzed by FACS in three independent experiments. Autofluorescence and relative cell size were used for normalizing bound fluorescence. Statistics: n.s: not significant, *, p < 0.05 (PDF) [file ppat.1010781.s003.pdf]
