# Supplementary material for: Genome wide CRISPR screen for Pasteurella multocida toxin (PMT) binding proteins reveals LDL Receptor Related Protein 1 (LRP1) as crucial cellular receptor
Source: PLoS Pathog. 2022 Dec 14;18(12):e1010781. doi: 10.1371/journal.ppat.1010781 (PMC9797058; doi:10.1371/journal.ppat.1010781)
Supplement: S2 Fig — (PDF) [file ppat.1010781.s002.pdf]

	Knocked out gene	function
1	Lrp1	LDL Receptor Related Protein1 , receptor for lipoproteins, toxins and viruses (endocytosis, transcytosis)
2	Dph1	Diphthamide synthesis
3	Gsdmcl1	Gasdermin domain-containing protein (uncharacterized)
4	Mical2	Microtubuli binding protein
5	Gse1	Genetic Suppressor Element 1 (uncharacterized)
6	Aatk	Apoptosis Associated Tyrosine Kinase
7	Lrrc16b	Capping protein (regulation of the cytoskeleton)
8	Trim66	tripartite motif containing 66, gene silencing
9	Dph5	Diphthamide synthesis
10	Clip1	links endocytic vesicles to microtubules
